# Diaqua­bis­(hydrogen tartrato)copper(II) dihydrate

**DOI:** 10.1107/S160053681002115X

**Published:** 2010-06-16

**Authors:** Mohammad T.M. Al-Dajani, Hassan H. Abdallah, Nornisah Mohamed, Madhukar Hemamalini, Hoong-Kun Fun

**Affiliations:** a School of Pharmaceutical Sciences, Universiti Sains Malaysia, 11800 USM, Penang, Malaysia; b School of Chemical Sciences, Universiti Sains Malaysia, 11800 USM, Penang, Malaysia; c X-ray Crystallography Unit, School of Physics, Universiti Sains Malaysia, 11800 USM, Penang, Malaysia

## Abstract

The title complex, [Cu(C_4_H_5_O_6_)_2_(H_2_O)_2_]·2H_2_O, contains a Cu^II^ ion lying on an inversion centre. The coordination geometry of the Cu^II^ ion is a distorted octa­hedron with four O atoms from two hydrogen tartrate ions occupying the equatorial positions and two O atoms from two coordinated water mol­ecules occupying the axial positions. In the crystal structure, inter­molecular O—H⋯O and C—H⋯O hydrogen bonds link the mol­ecules into a three-dimensional network.

## Related literature

For background to coordination polymers, see: Stang & Olenyuk (1997 ▶); Aakeroy & Seddon (1993 ▶); Munakata *et al.* (1999 ▶); Fujita *et al.* (1994 ▶); Hagrman *et al.* (1997 ▶). For the optical activity of tartaric acid, see: Synoradzki *et al.* (2008 ▶). For related structures, see: Jian *et al.* (2005 ▶). For the stability of the temperature controller used for the data collection, see: Cosier & Glazer (1986 ▶).
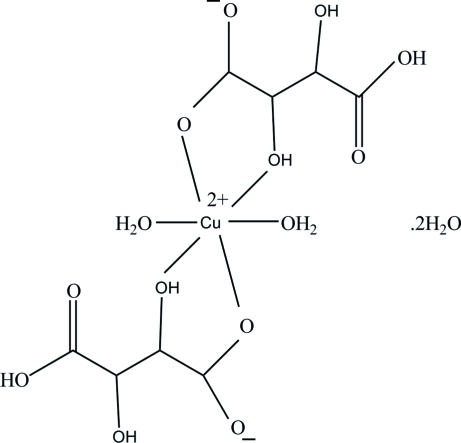


## Experimental

### 

#### Crystal data

[Cu(C_4_H_5_O_6_)_2_(H_2_O)_2_]·2H_2_O
*M* *_r_* = 433.76
Monoclinic, 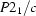

*a* = 7.1577 (8) Å
*b* = 14.0989 (14) Å
*c* = 7.8910 (8) Å
β = 109.136 (2)°
*V* = 752.32 (14) Å^3^
*Z* = 2
Mo *K*α radiation
μ = 1.54 mm^−1^
*T* = 100 K
0.42 × 0.15 × 0.08 mm

#### Data collection

Bruker APEXII DUO CCD area-detector diffractometer
Absorption correction: multi-scan (*SADABS*; Bruker, 2009 ▶) *T*_min_ = 0.563, *T*_max_ = 0.885
12361 measured reflections
3298 independent reflections
3001 reflections with *I* > 2σ(*I*)
*R*_int_ = 0.023

#### Refinement

*R*[*F* ^2^ > 2σ(*F* ^2^)] = 0.023
*wR*(*F* ^2^) = 0.082
*S* = 1.20
3298 reflections
121 parameters
H atoms treated by a mixture of independent and constrained refinement
Δρ_max_ = 0.68 e Å^−3^
Δρ_min_ = −0.45 e Å^−3^

### 

Data collection: *APEX2* (Bruker, 2009 ▶); cell refinement: *SAINT* (Bruker, 2009 ▶); data reduction: *SAINT*; program(s) used to solve structure: *SHELXTL* (Sheldrick, 2008 ▶); program(s) used to refine structure: *SHELXTL*; molecular graphics: *SHELXTL*; software used to prepare material for publication: *SHELXTL* and *PLATON* (Spek, 2009 ▶).

## Supplementary Material

Crystal structure: contains datablocks global, I. DOI: 10.1107/S160053681002115X/is2556sup1.cif

Structure factors: contains datablocks I. DOI: 10.1107/S160053681002115X/is2556Isup2.hkl

Additional supplementary materials:  crystallographic information; 3D view; checkCIF report

## Figures and Tables

**Table 1:** Hydrogen-bond geometry (Å, °)

*D*—H⋯*A*	*D*—H	H⋯*A*	*D*⋯*A*	*D*—H⋯*A*
O4—H4⋯O1*W*^i^	0.82	1.91	2.7200 (12)	170
O2—H5⋯O2*W*^ii^	0.72 (3)	1.82 (3)	2.5331 (12)	170 (3)
O6—H6⋯O3^iii^	0.82	1.70	2.5092 (12)	167
O1*W*—H11⋯O5^iv^	0.91	1.91	2.8119 (11)	175 (1)
O1*W*—H12⋯O4	0.96	1.85	2.8091 (11)	177
O2*W*—H21⋯O1^v^	0.90	1.94	2.8298 (12)	173
O2*W*—H22⋯O5^i^	0.89	1.98	2.8100 (12)	154
C2—H2⋯O6^ii^	0.98	2.43	3.2727 (12)	143
C3—H3⋯O4^i^	0.98	2.46	3.4160 (13)	166

Symmetry codes: (i) 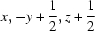; (ii) 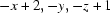; (iii) 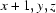; (iv) 
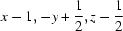
; (v) 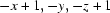.

**Fig. 1.**
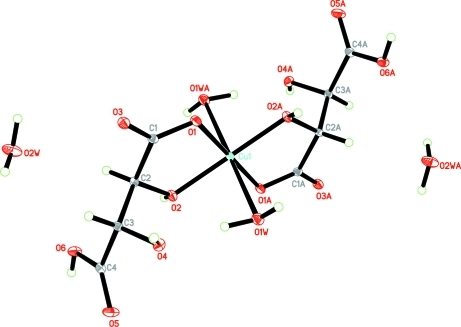
The molecular structure of the title compound, showing 50% probability displacement ellipsoids and the atom-numbering scheme. C1A–C4A/O1A–O6A/O1WA/O2WA are generated by the symmetry code 1-x, -y, -z.

**Fig. 2.**
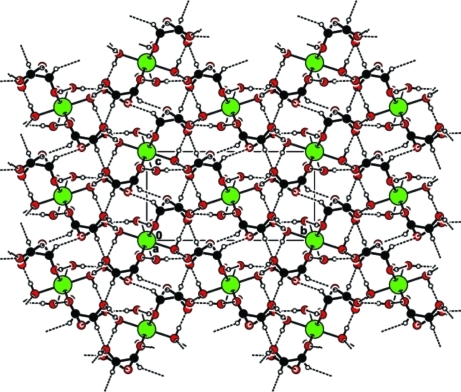
The crystal packing of the title compound, showing hydrogen-bonded (dashed lines) network.

## supplementary crystallographic information

### Comment

In recent years, there has been great interest in the study of coordination polymers with network structures due to their possible chemical and physical properties (Stang & Olenyuk, 1997; Aakeroy & Seddon, 1993; Munakata *et al.*, 1999). A number of unique networks have been obtained by reactions between transition metal ions and rationally designed organic ligands (Fujita *et al.*, 1994; Hagrman *et al.*, 1997). Tartaric acid has been used as building blocks to construct 1D, 2D and 3D frameworks due to the diversity of binding modes of the carboxyl group and hydroxyl group in the tartaric acid. It has many applications such as in making silver mirrors, in the manufacture of soft drinks, to provide tartness to foods, in tanning leather, and in making blueprints. Tartaric acid also has optical activity (Synoradzki *et al.*, 2008). We report the crystal structure of (I).

(I) consists of a copper ion lying on a crystallographic inversion centre, two hydrogen tartrate ions, two coordinated water molecules and two uncoordinated water molecules (Fig. 1). The environment about the copper(II) ion is a distorted octahedron with four oxygen atoms from two hydrogen tartrate ions and two oxygen atoms from the coordinated water molecules completing the coordination. All the four oxygen atoms from the two hydrogen tartrate anions occupy equatorial positions and the oxygen atoms from the water molecules occupy in the axial positions.  The equatorial and axial distances of Cu—O [Cu—O1 = 1.9327 (7) Å; Cu—O2 = 1.9637 (7) Å and Cu—O1W = 2.4651 (8) Å] agree with those reported for similar systems (Jian *et al.*, 2005).

In the crystal structure, intermolecular O1W—H12···O4, O4—H4···O1W, O2—H5···O2W, O6—H6···O3, O1W—H11···O5, O2W—H21···O1, O2W—H22···O5, C2—H2···O6 and C3—H3···O4 hydrogen bonds (Table 1) link the molecules into a three-dimensional network.

### Experimental

*DL*-Tartaric acid (0.02 mol, 3.0 g) was dissolved in distilled water in a flat bottom flask with magnetic stirrer. CuCl_2_ (0.01 mol, 1.45 g) was added in small portions with continuous stirring for three hours at room temperature. The blue crystals formed were washed with *N,N*-dimethylformamide then with methanol and dried at 353 K.

### Refinement

Atom H5 was located in a difference Fourier map and refined freely. The remaining H atoms, excepting the water H atoms, were positioned geometrically (C—H = 0.98 Å and O—H = 0.82 Å) and were refined using a riding model, with *U*_iso_(H) = 1.2*U*_eq_(C) or 1.5*U*_eq_(O). The water H atoms were located in the difference map and then treated as riding atoms on the parent O atoms, with O—H =  0.8936–0.9551 Å and *U*_iso_(H) = 1.5*U*_eq_(O).

### Crystal data

**Table:**

[Cu(C_4_H_5_O_6_)_2_(H_2_O)_2_]·2H_2_O	*F*(000) = 446
*M**_r_* = 433.76	*D*_x_ = 1.915 Mg m^−^^3^
Monoclinic, *P*2_1_/*c*	Mo *K*α radiation, λ = 0.71073 Å
Hall symbol: -P 2ybc	Cell parameters from 6852 reflections
*a* = 7.1577 (8) Å	θ = 3.6–35.0°
*b* = 14.0989 (14) Å	µ = 1.54 mm^−^^1^
*c* = 7.8910 (8) Å	*T* = 100 K
β = 109.136 (2)°	Plate, blue
*V* = 752.32 (14) Å^3^	0.42 × 0.15 × 0.08 mm
*Z* = 2	

### Data collection

**Table:**

Bruker APEXII DUO CCD area-detector diffractometer	3298 independent reflections
Radiation source: fine-focus sealed tube	3001 reflections with *I* > 2σ(*I*)
graphite	*R*_int_ = 0.023
φ and ω scans	θ_max_ = 35.0°, θ_min_ = 2.9°
Absorption correction: multi-scan (*SADABS*; Bruker, 2009)	*h* = −11→11
*T*_min_ = 0.563, *T*_max_ = 0.885	*k* = −21→22
12361 measured reflections	*l* = −12→12

### Refinement

**Table:**

Refinement on *F*^2^	Primary atom site location: structure-invariant direct methods
Least-squares matrix: full	Secondary atom site location: difference Fourier map
*R*[*F*^2^ > 2σ(*F*^2^)] = 0.023	Hydrogen site location: inferred from neighbouring sites
*wR*(*F*^2^) = 0.082	H atoms treated by a mixture of independent and constrained refinement
*S* = 1.20	*w* = 1/[σ^2^(*F*_o_^2^) + (0.0456*P*)^2^ + 0.1293*P*] where *P* = (*F*_o_^2^ + 2*F*_c_^2^)/3
3298 reflections	(Δ/σ)_max_ = 0.001
121 parameters	Δρ_max_ = 0.68 e Å^−^^3^
0 restraints	Δρ_min_ = −0.45 e Å^−^^3^

### Special details

**Table:**

Experimental. The crystal was placed in the cold stream of an Oxford Cryosystems Cobra open-flow nitrogen cryostat (Cosier & Glazer, 1986) operating at 100.0 (1) K.
Geometry. All s.u.'s (except the s.u. in the dihedral angle between two l.s. planes) are estimated using the full covariance matrix. The cell s.u.'s are taken into account individually in the estimation of s.u.'s in distances, angles and torsion angles; correlations between s.u.'s in cell parameters are only used when they are defined by crystal symmetry. An approximate (isotropic) treatment of cell s.u.'s is used for estimating s.u.'s involving l.s. planes.
Refinement. Refinement of F^2^ against ALL reflections. The weighted R-factor wR and goodness of fit S are based on F^2^, conventional R-factors R are based on F, with F set to zero for negative F^2^. The threshold expression of F^2^ > 2σ(F^2^) is used only for calculating R-factors(gt) etc. and is not relevant to the choice of reflections for refinement. R-factors based on F^2^ are statistically about twice as large as those based on F, and R- factors based on ALL data will be even larger.

### Fractional atomic coordinates and isotropic or equivalent isotropic displacement parameters (Å^2^)

**Table:**

	*x*	*y*	*z*	*U*_iso_*/*U*_eq_	
Cu1	0.5000	0.0000	0.0000	0.00746 (6)	
O1	0.39754 (10)	0.03576 (5)	0.18906 (9)	0.00912 (12)	
O2	0.75259 (11)	0.04633 (5)	0.16577 (9)	0.00829 (12)	
O3	0.48127 (11)	0.11064 (5)	0.45343 (10)	0.00963 (12)	
O4	0.69066 (11)	0.24378 (5)	0.22934 (10)	0.00920 (12)	
H4	0.6209	0.2764	0.2705	0.014*	
O5	1.08144 (12)	0.25669 (5)	0.29938 (12)	0.01450 (14)	
O6	1.13354 (11)	0.12193 (5)	0.45912 (10)	0.01096 (13)	
H6	1.2407	0.1230	0.4424	0.016*	
C1	0.52178 (13)	0.07526 (6)	0.32500 (12)	0.00733 (14)	
C2	0.73709 (13)	0.08036 (6)	0.33188 (11)	0.00687 (14)	
H2	0.8181	0.0405	0.4303	0.008*	
C3	0.80915 (13)	0.18289 (6)	0.36467 (12)	0.00714 (14)	
H3	0.8008	0.2036	0.4805	0.009*	
C4	1.02337 (14)	0.19094 (6)	0.37032 (12)	0.00806 (14)	
O1W	0.46452 (11)	0.16624 (5)	−0.10244 (10)	0.01087 (13)	
H11	0.3385	0.1878	−0.1356	0.016*	
H12	0.5389	0.1917	0.0120	0.016*	
O2W	0.94287 (12)	0.05593 (6)	0.78307 (13)	0.01680 (16)	
H21	0.8316	0.0315	0.7943	0.025*	
H22	0.9492	0.1191	0.7909	0.025*	
H5	0.832 (5)	0.0119 (17)	0.174 (4)	0.034 (7)*	

### Atomic displacement parameters (Å^2^)

**Table:**

	*U*^11^	*U*^22^	*U*^33^	*U*^12^	*U*^13^	*U*^23^
Cu1	0.00525 (8)	0.00959 (9)	0.00761 (8)	−0.00150 (5)	0.00221 (6)	−0.00224 (4)
O1	0.0068 (3)	0.0113 (3)	0.0093 (3)	−0.0019 (2)	0.0027 (2)	−0.0022 (2)
O2	0.0063 (3)	0.0096 (3)	0.0089 (3)	0.0004 (2)	0.0025 (2)	−0.0024 (2)
O3	0.0082 (3)	0.0113 (3)	0.0103 (3)	−0.0005 (2)	0.0042 (2)	−0.0021 (2)
O4	0.0084 (3)	0.0086 (3)	0.0103 (3)	0.0030 (2)	0.0026 (2)	0.0020 (2)
O5	0.0092 (3)	0.0115 (3)	0.0228 (4)	−0.0008 (2)	0.0052 (3)	0.0063 (3)
O6	0.0064 (3)	0.0098 (3)	0.0168 (3)	0.0018 (2)	0.0041 (2)	0.0043 (2)
C1	0.0068 (3)	0.0063 (3)	0.0089 (3)	0.0001 (3)	0.0026 (3)	0.0008 (3)
C2	0.0055 (3)	0.0071 (3)	0.0080 (3)	−0.0003 (2)	0.0021 (3)	−0.0006 (3)
C3	0.0061 (3)	0.0068 (3)	0.0083 (3)	0.0001 (3)	0.0019 (3)	0.0002 (2)
C4	0.0071 (3)	0.0073 (3)	0.0093 (3)	−0.0003 (3)	0.0021 (3)	−0.0010 (3)
O1W	0.0090 (3)	0.0101 (3)	0.0127 (3)	0.0002 (2)	0.0024 (2)	0.0003 (2)
O2W	0.0104 (3)	0.0101 (3)	0.0321 (4)	0.0008 (2)	0.0098 (3)	0.0007 (3)

### Geometric parameters (Å, °)

**Table:**

Cu1—O1	1.9327 (7)	O5—C4	1.2239 (12)
Cu1—O1^i^	1.9327 (7)	O6—C4	1.3027 (11)
Cu1—O2^i^	1.9637 (7)	O6—H6	0.8200
Cu1—O2	1.9637 (7)	C1—C2	1.5259 (13)
Cu1—O1W	2.4651 (8)	C2—C3	1.5281 (13)
Cu1—O1W^i^	2.4651 (8)	C2—H2	0.9800
O1—C1	1.2753 (11)	C3—C4	1.5235 (13)
O2—C2	1.4339 (11)	C3—H3	0.9800
O2—H5	0.73 (3)	O1W—H11	0.9051
O3—C1	1.2461 (11)	O1W—H12	0.9551
O4—C3	1.4152 (11)	O2W—H21	0.8982
O4—H4	0.8200	O2W—H22	0.8936
			
O1—Cu1—O1W	88.90 (3)	O3—C1—C2	116.99 (8)
O1—Cu1—O1W^i^	91.11 (3)	O1—C1—C2	117.92 (8)
O1W—Cu1—O2	82.85 (3)	O2—C2—C1	109.36 (7)
O1W—Cu1—O1W^i^	180	O2—C2—C3	110.39 (7)
O1W—Cu1—O2	82.85 (3)	C1—C2—C3	109.36 (7)
O1W^i^—Cu1—O2^i^	82.85 (3)	O2—C2—H2	109.2
O1^i^—Cu1—O1W^i^	88.90 (3)	C1—C2—H2	109.2
O1—Cu1—O1^i^	180.00 (6)	C3—C2—H2	109.2
O1—Cu1—O2^i^	95.83 (3)	O4—C3—C4	108.99 (7)
O1^i^—Cu1—O2^i^	84.17 (3)	O4—C3—C2	111.13 (7)
O1—Cu1—O2	84.17 (3)	C4—C3—C2	110.86 (7)
O1^i^—Cu1—O2	95.83 (3)	O4—C3—H3	108.6
O2^i^—Cu1—O2	180.0	C4—C3—H3	108.6
C1—O1—Cu1	115.26 (6)	C2—C3—H3	108.6
C2—O2—Cu1	112.96 (5)	O5—C4—O6	125.12 (9)
C2—O2—H5	116 (2)	O5—C4—C3	122.17 (8)
Cu1—O2—H5	111 (2)	O6—C4—C3	112.71 (8)
C3—O4—H4	109.5	H11—O1W—H12	110.0
C4—O6—H6	109.5	H21—O2W—H22	113.7
O3—C1—O1	125.09 (9)		
			
O2^i^—Cu1—O1—C1	−176.70 (7)	O3—C1—C2—O2	−173.54 (8)
O2—Cu1—O1—C1	3.30 (7)	O1—C1—C2—O2	6.20 (11)
O1—Cu1—O2—C2	0.36 (6)	O3—C1—C2—C3	−52.55 (10)
O1^i^—Cu1—O2—C2	−179.64 (6)	O1—C1—C2—C3	127.19 (8)
O1W—Cu1—O1—C1	−79.64 (6)	O2—C2—C3—O4	62.36 (9)
O1W^i^—Cu1—O1—C1	100.37 (6)	C1—C2—C3—O4	−58.01 (9)
O1W—Cu1—O2—C2	89.98 (6)	O2—C2—C3—C4	−59.01 (9)
O1W^i^—Cu1—O2—C2	−90.02 (6)	C1—C2—C3—C4	−179.37 (7)
Cu1—O1—C1—O3	173.56 (7)	O4—C3—C4—O5	16.25 (12)
Cu1—O1—C1—C2	−6.17 (10)	C2—C3—C4—O5	138.88 (9)
Cu1—O2—C2—C1	−3.22 (9)	O4—C3—C4—O6	−164.52 (8)
Cu1—O2—C2—C3	−123.59 (6)	C2—C3—C4—O6	−41.90 (10)

Symmetry codes: (i) −*x*+1, −*y*, −*z*.

### Hydrogen-bond geometry (Å, °)

**Table:**

*D*—H···*A*	*D*—H	H···*A*	*D*···*A*	*D*—H···*A*
O4—H4···O1W^ii^	0.82	1.91	2.7200 (12)	170
O2—H5···O2W^iii^	0.72 (3)	1.82 (3)	2.5331 (12)	170 (3)
O6—H6···O3^iv^	0.82	1.70	2.5092 (12)	167
O1W—H11···O5^v^	0.91	1.91	2.8119 (11)	175 (1)
O1W—H12···O4	0.96	1.85	2.8091 (11)	177
O2W—H21···O1^vi^	0.90	1.94	2.8298 (12)	173
O2W—H22···O5^ii^	0.89	1.98	2.8100 (12)	154
C2—H2···O6^iii^	0.98	2.43	3.2727 (12)	143
C3—H3···O4^ii^	0.98	2.46	3.4160 (13)	166

Symmetry codes: (ii) *x*, −*y*+1/2, *z*+1/2; (iii) −*x*+2, −*y*, −*z*+1; (iv) *x*+1, *y*, *z*; (v) *x*−1, −*y*+1/2, *z*−1/2; (vi) −*x*+1, −*y*, −*z*+1.
